# Unresectable Extraskeletal Myxoid Chondrosarcoma of the Neck: Early Tumor Response to Chemoradiotherapy

**DOI:** 10.7759/cureus.432

**Published:** 2015-12-24

**Authors:** Mark Zaki, Pam Laszewski, Natasha Robinette, Husain Saleh, Naweed Raza, Ammar Sukari, Harold Kim

**Affiliations:** 1 Radiation Oncology, Karmanos Cancer Center; 2 Diagnostic Radiology, Karmanos Cancer Center; 3 Pathology, Detroit Medical Center; 4 Otolaryngology, Karmanos Cancer Center; 5 Medical Oncology, Karmanos Cancer Center

**Keywords:** soft tissue sarcoma, radiation oncology, chemotherapy, head and neck pathology

## Abstract

Extraskeletal myxoid chondrosarcoma (EMC) rarely occurs in the head and neck and is generally managed with primary surgery. To our knowledge, no cases of unresectable EMC of the neck have been reported. We present a case of an unresectable EMC treated with chemotherapy and radiation, and highlight the exceptional early response to therapy.

## Introduction

Extraskeletal myxoid chondrosarcoma (EMC) is a rare, soft-tissue tumor considered to be low-grade, with a slow growth pattern and prolonged clinical course, yet it has a high rate of local recurrence and metastasis [1]. It generally arises from the deep soft tissues of proximal limbs, trunk, or buttocks, though rare cases have been reported to occur in other body sites [1]. Few cases have been reported to occur in the neck [2-5]. There are limited data on the treatment of this rare malignancy. In general, EMC is thought to be resistant to both chemotherapy and radiation; therefore, surgical resection is the most commonly employed treatment option [1]. We present a case of rapidly growing EMC of the neck with an exceptional early response to chemoradiation therapy.

## Case presentation

A 65-year-old male presented with an eight-week history of a left-sided neck mass. The patient described a mass, which was initially the size of a golf ball, but grew rapidly. By the second month, it caused significant pain, dysphagia, and dyspnea, prompting an urgent visit to the hospital. Pertinent physical examination findings at presentation included a firm, large 20 cm mass which occupied the entire left neck. The mass crossed the midline, displacing the trachea to the right side. 

**Figure 1:**
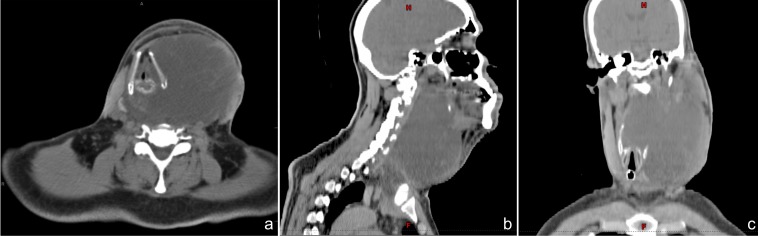
Pre-Treatment CT Scan Initial (a) axial, (b) saggital, and (c) coronal simulation CT scan revealing a large (9.5 cm x 12.9 cm x 21 cm) infiltrative mass involving the fascial spaces of the left neck and the anterior and superior mediastinum.

The patient was admitted and a computed-tomography (CT) scan was performed (Figure 1) revealing a large (9.5 cm x 12.9 cm x 21 cm), trans-spatial, infiltrative, mixed solid and cystic mass involving the left carotid, parotid, masticator, parapharygeal, and prevertebral spaces. The tumor extended through the left thyrohyoid membrane and involved the submucosal surface of the oropharynx, hypopharynx, and larynx and crossed the midline via the retropharyngeal space, insinuating between the right thyroid lobe and cricoid cartilage and extended inferiorly into the anterior and superior mediastinum. There was also severe narrowing of the supraglottic airway and rightward deviation of the entire laryngeal axis. No obvious erosion of adjacent osseous structures, hyoid bone, laryngeal cartilage, or tracheal cartilaginous rings was appreciated.

**Figure 2:**
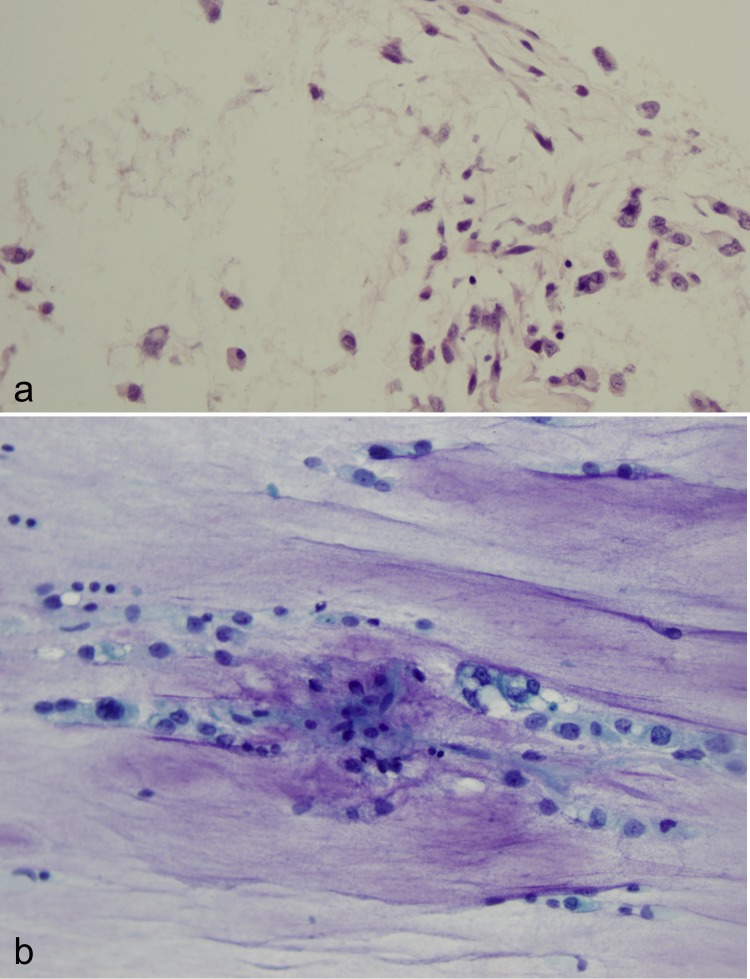
Pathology (a) Core biopsy of the tumor shows cords and strands of small, cohesive, eosinophilic cells immersed in a large amount of myxoid matrix. This is the classic histologic finding in extraskeletal myxoid chondrosarcoma. (b) Areas composed of large cells with pale vesicular nuclei and prominent nucleoli are present. Substantial cytoplasmic vacuolization is seen and mitotic figures are readily identified within the cells.

A tracheostomy tube and a percutaneous feeding tube were placed. Additionally, a biopsy of the mass was performed. This revealed a myxoid sarcoma composed of cords, clusters, and strands of cohesive epithelioid cells within a large amount of myxoid matrix. Pale vesicular nuclei and mitotic figures were readily identified within the cells. Immunohistochemistry (IHC) stains were negative for brachyury, cytokeratin, smooth muscle actin, and S100. Based on the pathologic appearance of the cells and IHC features, the diagnosis of extraskeletal myxoid chondrosarcoma with prominent cytoplasmic vacuolization was established (Figure 2).

**Figure 3:**
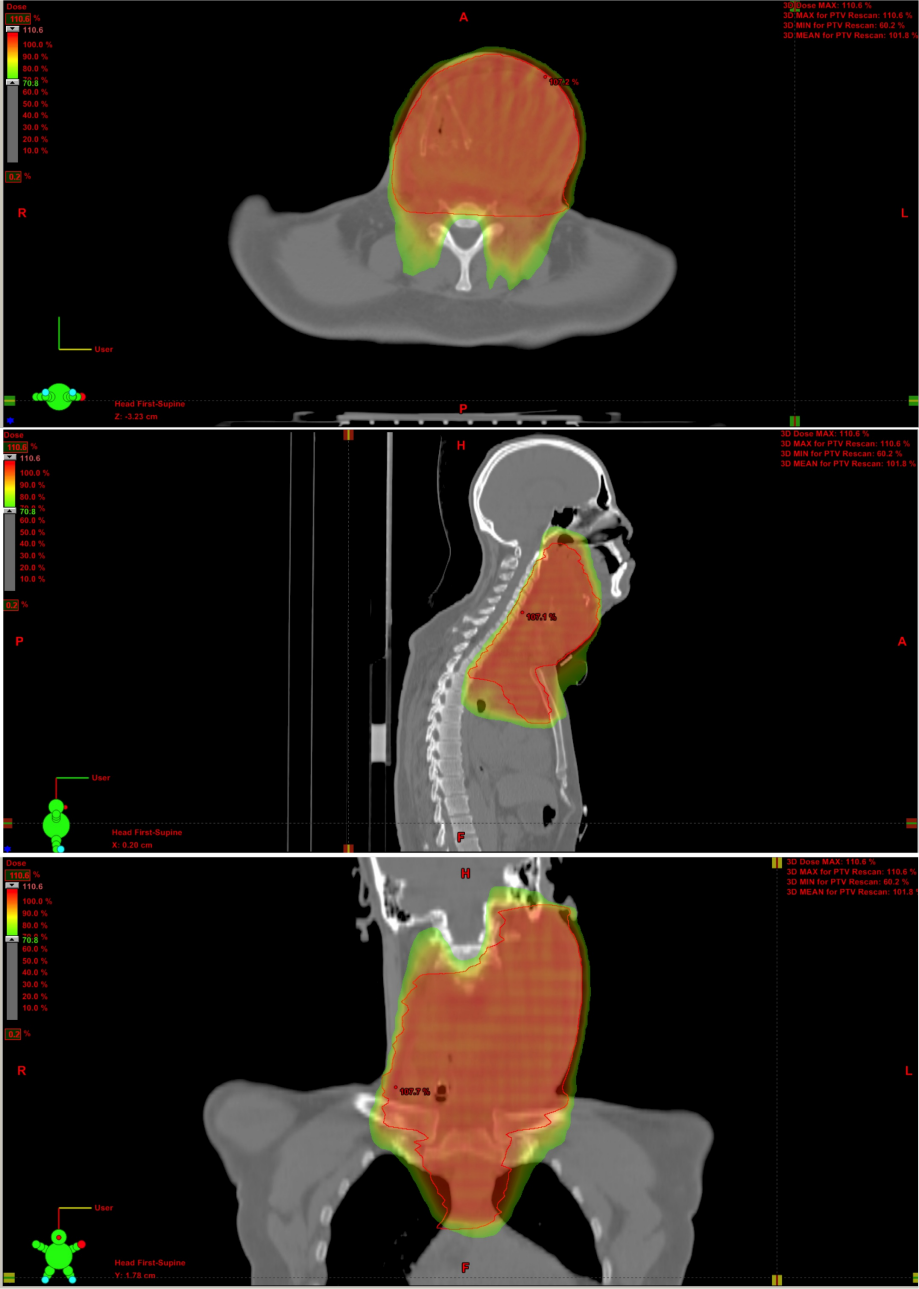
Radiation Treatment Plan Initial radiation treatment plan: solid red line = planning target volume (PTV); prescription dose = 7000 cGy in 35 fractions. Dose color wash indicates area receiving the prescription dose.

The tumor was considered to be unresectable due to its locally advanced nature.  The patient was recommended to receive concurrent chemotherapy and radiation. Informed patient consent was obtained. He was treated with eight weekly cycles of doxorubicin (10 mg/m^2^) with concurrent image-guided, intensity modulated radiotherapy to a dose of 7000 centigray (cGy) in 35 fractions given over the course of seven weeks (Figure 3).

**Figure 4:**
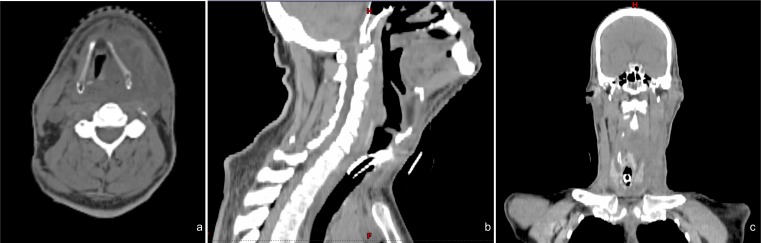
Repeat Simulation CT Scan Repeat (a) axial, (b) saggital, and (c) coronal simulation CT scan after 4400 cGy showing dramatic tumor response to chemoradiation therapy. Tumor measures 5.2 cm x 6.6 cm x 14 cm 30 days after the start of therapy.

During treatment, he was noted to have a drastic response to therapy, necessitating re-simulation and re-planning of radiotherapy after 4400 cGy (Figure 4).  At the completion of the concurrent therapy, the patient was noted to have a remarkable clinical response with a significant improvement in speech and swallowing and in tumor size and pain.

He tolerated therapy fairly well and completed the planned treatment with no breaks. He experienced the expected side effects of grade 3 mucositis and grade 3 radiation dermatitis (Common Terminology Criteria for Adverse Events version 4.0), which were managed with opioid analgesics, topical ointments, and silver sulfadiazine cream. Near the end of the therapy, he was suspected to have superimposed cellulitis that was successfully treated with antibiotics.

During the course of the concurrent chemoradiotherapy, molecular profiling (Caris Life Sciences, Phoenix, AZ) was performed on the patient’s tumor for biomarkers of drug response. High expression of TLE3 and negative IHC staining for PGP and TUBB3 suggested a potential benefit for docetaxel, paclitaxel, or nab-paclitaxel. IHC staining was negative for ERCC1, suggesting a potential benefit for carboplatin, cisplatin, or oxaliplatin.

**Figure 5:**
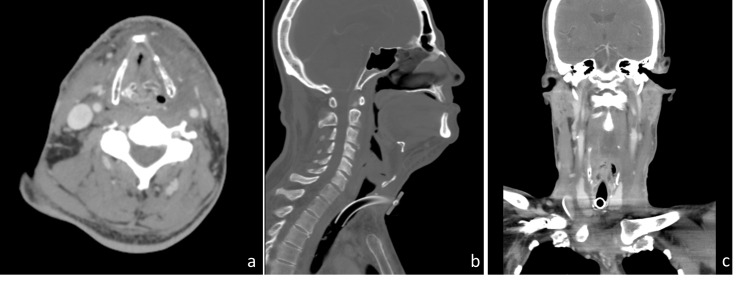
One Month Follow-up CT Scan Follow-up (a) axial, (b) saggital, and (c) coronal CT scan one month after the completion of radiation. Diffuse mucosal edema and enhancement in the pharyngeal mucosal space, oral cavity, and larynx are related to postradiation changes. Previously seen large hypodense mass centered in the left neck has significantly decreased in size.

One month after the completion of therapy, the patient is recovering as expected from the acute toxicity of therapy and is demonstrating a complete clinical response and an excellent radiographic response (Figure 5). Further chemotherapy with carboplatin and paclitaxel is planned given the results of molecular profiling.

## Discussion

To our knowledge, only three previous case reports have described EMC of the neck [2-5].  In all of these reports, the primary treatment was surgical resection. A unique challenge was faced in this case as the tumor was not amenable to resection; thus, we elected to proceed with chemoradiation.

Despite the paucity of data, EMC is generally regarded to respond poorly to chemotherapy or radiation. In this case, the chemotherapy regimen was selected in part based on a small series of patients with locally advanced or metastatic EMC treated with anthracycline-based chemotherapy. In this series, 70% of patients had either a partial response or stable disease [6]. The schedule and dose of chemotherapy were chosen with the aim of attaining the radiosensitizing effect of concurrent low-dose doxorubicin as established in the treatment of anaplastic thyroid cancer [7]. The radiotherapy dose of 7000 cGy was based on published experience with soft tissue sarcomas and chondrosarcoma of the bone [8] and the known normal tissue tolerances of other organs in the treatment field.

EMC has been described in many reports as having an indolent clinical course. The presented case is rare in that the patient had rapid clinical progression prior to therapy. The mechanism responsible for this accelerated growth rate is unknown. It may be hypothesized that certain genetic, biologic, or molecular variables underlie the observed quick tumor growth, as well as the rapid early tumor response to treatment.  

Though sarcomas are categorically classified as radioresistant, there are data showing that myxoid liposarcomas are highly sensitive to radiotherapy [9]. In de Vreeze's series, eight patients with pure myxoid liposarcoma achieved a pathologic complete response, and approximately half of the other sarcomas showed 80% to 90% pathologic response after preoperative radiotherapy. The authors noted that these radiosensitive specimens contained branching vasculature, partial thrombus formation, and inflammation of medium-sized arterioles, similar to the vascular changes in myxoid liposarcoma. In our patient, there was an abundance of vascular channels surrounding the malignant cells. This pathologic feature is a plausible reason for the substantial clinical response observed.

The radiographic appearance of the tumor may also lend insight to the favorable response to chemoradiotherapy. Though quite large, the tumor had a growth pattern that was progressively insinuating into various compartments of the neck and mediastinum, rather than direct invasion into adjacent structures. This feature may indicate less invasive potential of the tumor, which could relate to greater susceptibility to cytotoxic therapy [10].

## Conclusions

We present a case of unresectable EMC of the neck with excellent, early response to combined chemoradiotherapy. Further follow-up is necessary to assess the durability of response; yet, this approach may prove to be a viable strategy in unresectable cases. 
